# Yimusake ameliorates corporal endothelial dysfunction by down-regulating the NLRP3 inflammasome–mediated NF-κB signaling pathway and inhibiting oxidative stress

**DOI:** 10.1093/sexmed/qfaf079

**Published:** 2025-10-13

**Authors:** Chengxia Yang, Rui Zhang, Bingbing Zhu, Lipan Niu, Wenfei Wang, Xiufang Jin, Yulian Liu, Fengxia Liu

**Affiliations:** Department of Human Anatomy, College of Basic Medical Sciences, Xinjiang Medical University, Urumqi, Xinjiang Uygur Autonomous Region 830011, China; Xinjiang Key Laboratory of Molecular Biology of Endemic Diseases, Urumqi, Xinjiang Uygur Autonomous Region 830011, China; Department of Human Anatomy, College of Basic Medical Sciences, Xinjiang Medical University, Urumqi, Xinjiang Uygur Autonomous Region 830011, China; Xinjiang Key Laboratory of Molecular Biology of Endemic Diseases, Urumqi, Xinjiang Uygur Autonomous Region 830011, China; Department of Human Anatomy, College of Basic Medical Sciences, Xinjiang Medical University, Urumqi, Xinjiang Uygur Autonomous Region 830011, China; Xinjiang Key Laboratory of Molecular Biology of Endemic Diseases, Urumqi, Xinjiang Uygur Autonomous Region 830011, China; Department of Human Anatomy, College of Basic Medical Sciences, Xinjiang Medical University, Urumqi, Xinjiang Uygur Autonomous Region 830011, China; Xinjiang Key Laboratory of Molecular Biology of Endemic Diseases, Urumqi, Xinjiang Uygur Autonomous Region 830011, China; College of Clinical Medicine, Xinjiang Medical University, Urumqi, Xinjiang Uygur Autonomous Region 830011, China; Department of Human Anatomy, College of Basic Medical Sciences, Xinjiang Medical University, Urumqi, Xinjiang Uygur Autonomous Region 830011, China; Xinjiang Key Laboratory of Molecular Biology of Endemic Diseases, Urumqi, Xinjiang Uygur Autonomous Region 830011, China; Department of Human Anatomy, College of Basic Medical Sciences, Xinjiang Medical University, Urumqi, Xinjiang Uygur Autonomous Region 830011, China; Xinjiang Key Laboratory of Molecular Biology of Endemic Diseases, Urumqi, Xinjiang Uygur Autonomous Region 830011, China; Department of Human Anatomy, College of Basic Medical Sciences, Xinjiang Medical University, Urumqi, Xinjiang Uygur Autonomous Region 830011, China; Xinjiang Key Laboratory of Molecular Biology of Endemic Diseases, Urumqi, Xinjiang Uygur Autonomous Region 830011, China

**Keywords:** diabetes mellitus–induced erectile dysfunction, NLRP3 inflammasome–mediated NF-κB signaling pathway, Yimusake, oxidative stress, endothelial dysfunction

## Abstract

**Background:**

Yimusake, a traditional Uyghur medicine, can treat a variety of male diseases, but its effects and mechanisms on diabetes mellitus–induced erectile dysfunction (DMED) remain unclear.

**Aim:**

This study was designed to investigate the key cytokines and mechanisms by which Yimusake ameliorates corporal endothelial dysfunction in DMED rats.

**Methods:**

Firstly, normal rat penile corpus cavernosum endothelial cells (CCECs) were extracted and cultured *in vitro*, and the injury model was established after stimulation with 30 mM glucose for 24 hours. Subsequently, the cells were cultured in Yimusake drug-containing serum for 24 hours, and Sh-NLRP3 lentivirus was transfected for 8 hours. Cells were collected for the subsequent experiments. Next, the DM model was established using streptozotocin (45 mg/kg) for 2 consecutive injections, and DMED rats were screened by apomorphine and mating test at weeks 2, 4, 6, and 8, and then, we intervened for a fortnight using Yimusake (Y) and NLRP3 inhibitor (MCC950) drugs, and the penile tissues were taken for the subsequent analyses.

**Outcomes:**

Our study demonstrates that Yimusake can downregulate the NLRP3-mediated NF-κB signalling pathway, inhibiting oxidative stress and mitigating the endothelial damage in corpus cavernosum endothelial cells.

**Results:**

In the CCEC injury model established by 30 mM glucose, after incubation with Yimusake-containing drug serum and transfection with Sh-NLRP3 lentivirus, the expression of proteins and mRNAs related to the NF-κB signaling pathway mediated by NLRP3 was decreased, the expression of proteins related to oxidative stress was decreased, and the expression of proteins related to the endothelial function was increased. In DMED rats, after Yimusake and MCC950 interventions, the changes in pathway factors, oxidative stress levels, and endothelial function were consistent with the trends of *in vitro* experiments.

**Clinical Implications:**

Yimusake may ameliorate endothelial dysfunction in DMED by down-regulating the NLRP3 inflammasome–mediated NF-κB signaling pathway and inhibiting oxidative stress.

**Strengths and Limitations:**

Although we revealed that Yimusake can facilitate the restoration of the erectile tissue by improving endothelial function through inhibiting inflammation and oxidative stress, the effect was more significant with the combination of drugs, and the exact mechanism of action needs to be further explored.

**Conclusion:**

These findings demonstrate that DM exacerbates oxidative stress and endothelial damage in the corpus cavernosum through activation of the NLRP3 inflammasome–mediated NF-κB pathway, whereas Yimusake can inhibit oxidative stress and mitigate endothelial damage by downregulating this pathway, thereby facilitating the restoration of erectile tissue in rats with DMED.

## Introduction

Erectile dysfunction (ED), as one of the most prevalent complications in diabetic men, is characterized by the inability to maintain satisfactory sexual intercourse and the significant impacts on patients’ physical and mental health, as well as their overall quality of life.^1^ The incidence of ED in diabetic individuals ranges from 37.5% to 66.3%, which is 3.5 times higher than the general population.^2^ Diabetes mellitus–induced erectile dysfunction (DMED) is characterized by neurovascular penile lesions that result in a poor response to conventional ED treatments, such as PDE5 inhibitors. Therefore, it is imperative to investigate novel therapeutic approaches for the management of DMED.

The mechanism of DMED is multifaceted and frequently involves structural alterations in penile vasculature, neuroendocrine disturbances, and other contributing factors.^3^ Corpus cavernosum endothelial cells (CCECs), which line the surface of the penile sinus cavities, perform a variety of physiological functions, including the synthesis of vasodilator and vasoconstrictor factors and the formation of blood vessel walls, playing a crucial role in the penile erection process.^4^ Research has shown that high glucose levels can reduce the activity of endothelial nitric oxide synthase (eNOS) in CCECs, leading to decreased nitric oxide (NO) release by endothelial cells. Consequently, this process can induce structural alterations in penile vasculature, resulting in vascular endothelial dysfunction. This dysfunction can restrict the dilation of sinusoidal vessels, reduce blood flow, and ultimately impair penile erectile function.^5^ Therefore, endothelial dysfunction serves as the pathophysiological basis for the development of DMED, and early intervention for diabetes-induced endothelial dysfunction may delay the onset and progression of DMED. In recent years, numerous studies have explored the mechanisms of endothelial dysfunction in DMED and have identified a strong association with inflammation and oxidative stress. Hyperglycemia can induce the accumulation of reactive oxygen species (ROS), and the continuous accumulation of ROS induces inflammatory damage in endothelial cells by regulating the expression of inflammation-related factors, ultimately leading to DMED.^6^ NOD-like receptor thermal protein domain associated protein 3 (NLRP3) inflammasomes, a cytoplasmic multiprotein complex, play a crucial role in the stimulation and activation of inflammation.^7^ As a transcription factor, nuclear factor kappa-B (NF-κB) plays a critical role in regulating genes associated with inflammatory responses. Upregulation of inflammatory cytokines can further amplify the activation of the NF-κB signaling pathway.^8^ Moreover, NLRP3 inflammasomes can regulate the inflammatory response by promoting NF-κB activation.^9^^,^^10^ Studies have demonstrated decreased expression of anti-inflammatory cytokines, elevated oxidative stress, and increased endothelial cell apoptosis in the penile tissue of diabetic mice with DMED, collectively contributing to erectile dysfunction.^11^ Although inflammation and oxidative stress are established pathogenic factors in DMED, the precise molecular mechanisms underlying endothelial injury remain unclear.

Our previous study focused on the pyroptosis and necroptosis pathways in DMED,^12^^,^^13^ whereas the current work specifically explores the role of the NLRP3 inflammasome–mediated NF-κB pathway and oxidative stress in endothelial dysfunction, providing a new mechanistic perspective for this field.

Yimusake, a traditional Uyghur medicine, is renowned for its kidney-nourishing and yang-invigorating properties, which enhance sperm quality and strengthen the body’s essence. It is composed of11 medicinal materials including *Bletilla striata*, artificial musk, ambergris, *Crocus sativus*, *Strychnos nux-vomica*, frankincense, bull penis, *Myristica fragrans*, *Syzygium aromaticum*, *Papaver somniferum*, and *Alpinia officinarum*. It is commonly prescribed for conditions such as impotence, premature ejaculation, spermatorrhea, enuresis, and neurasthenia.^14^ The therapeutic mechanism of Yimusake involves improving endothelial function and increasing testosterone levels by modulating the gonadal axis, activating nitric oxide/guanosine cyclic phosphate and vasoactive intestinal peptide/adenosine cyclic phosphate, etc.^15^ Does Yimusake inhibit oxidative stress, improve endothelial dysfunction and promote erectile tissue recovery in DMED rats by down-regulating the NLRP3 inflammasome-mediated NF-κB signaling pathway? This study aims to test this hypothesis and clarify the underlying mechanism by investigating the effects of Yimusake on DMED rats through the NLRP3 inflammasome–mediated NF-κB pathway.

## Materials and methods

### C‌CEC extraction, culture, and infection

Consistent with previous research methods,^16^ CCECs were isolated and cultured in ECM medium (1001, Science Cell, USA) and incubated at 37 °C and 5% CO_2_. The cells were subcultured to the second to fourth generation using trypsin (S6070, GE-HYCLONE, USA) for subsequent experimental procedures.

The Lentivirus containing negative control short hairpin RNA (Sh-Ctrl) and Sh-RNA targeting NLRP3 (Sh-NLRP3) was constructed by GeneChem (GCD0328528, Genechem, China). Corpus cavernosum endothelial cells were planted into 6-well plates for 24 hours. After 24 hours, the lentiviruses were added and incubated for 8 hours and then transferred to the complete ECM medium. Subsequently, the efficiency of infection was determined by quantitative real-time PCR and Western blot.

The cells were divided into the following groups: NC group, high glucose (HG) group, Sh-Ctrl group, Sh-NLRP3 group, Yimusake (Z65020144, Hetian Uyghur Pharmaceutical, China) drug-containing serum (Y) group, and Sh-NLRP3 + Y group. Except for the NC group, all other groups were exposed to 30 mM glucose stimulation for 24 hours. The Yimusake drug–containing serum was derived from 6-week-old male Sprague–Dawley rats that had received Yimusake via gastric gavage at a dose of 250 mg/kg for 1 week. The theory of serum pharmacochemistry posits that only components that enter the bloodstream can exert pharmacological effects.^17^ Traditional Chinese medicine, known for its complex composition, must be digested and absorbed to reach target organs via blood circulation,^18^ which presents a challenge for cellular-level studies. To address this, we utilized serum from Sprague-Dawley (SD) rats post-gavage to preserve the integrity and synergistic effects inherent in herbal treatments,^19^ thereby more accurately reflecting the true response of Yimusake within the rat’s physiological environment. Subsequently, the serum obtained was employed for the following experiments.

### Immunofluorescence staining

After obtaining the rat penile tissue and the fixed CCECs, standard procedures including paraffin embedding, sectioning, dewaxing, and antigen retrieval were performed. Subsequently, rabbit anti-eNOS (dilution 1:200, catalog number AF0096, Affinity, China), CD31 (dilution 1:200, catalog number AF6191, Affinity, China), and von Willebrand Factor (vWF) (dilution 1:200, catalog number AF3000, Affinity, China) primary antibodies were applied to the samples and incubated overnight at 4 °C. The next day, after rewarming, fluorescent anti-rabbit antibodies (ab6939, abcam, USA) were added and incubated in the dark for 2 hours. This was followed by DAPI (C0065, Solarbio Science, China) nuclear staining for 5 minutes and sealing of the slides with 100 μL of glycerol (1280, BioFroxx, Germany). Finally, fluorescence intensity was observed and captured using a fluorescence inverted microscope (DMi8, Leica, Germany), with analysis performed using Image J.

### Methyl thiazolyl tetrazolium (MTT)

Corpus cavernosum endothelial cells were seeded in a 96-well plate, and upon complete cell adhesion, 20 μL of 5 mg/mL MTT solution (M7007, Abmole, USA) was added. After 4 hours, the old culture medium was removed, and 150 μL of dimethyl sulfoxide (1084, BioFroxx Germany) was added. Subsequently, the plate was placed in darkness on a shaker for 15 minutes, followed by OD value measurements at 490 nm using a microplate reader (xMark, Bio-Rad Laboratories, USA) to assess cell viability. The cell viability was calculated using the formula: cell viability = (OD value of the treated group − OD value of the cell-free well)/(OD value of the control group − OD value of the cell-free well), followed by statistical analysis.

### Scratch assay

Corpus cavernosum endothelial cells were cultivated in a 96-well plate until reaching approximately 80% confluency. A straight line was inscribed using a vertical 200 μL sterile pipette tip on the plate’s surface. The culture medium was then substituted with serum-free ECM, and the plate was transferred to a CO_2_ incubator (Thermo371, Thermo, USA) set at 37 °C. The cells were observed at 0-, 12-, and 24-hour intervals; the scratch width was measured under a microscope, documented via photography, and analyzed using Image J software.

### Kits to detect NO (S0021S, Beyotime, China) levels

Cells were inoculated into 6-well plates in advance, and when 80% confluent, adherent cells were digested, centrifuged at 1000 g for 10 minutes at 4 °C using a refrigerated centrifuge (H1750R, Hunan Xiangyi Laboratory Instrument Development, China), and the supernatant was collected. A Nitric Oxide Assay Kit was used for detection. Standards were prepared at concentrations of 0, 1, 2, 5, 10, 20, 40, 60, and 100 μM, with 50 μL added to standard wells; 50 μL of sample supernatant was added to sample wells. Then, 50 μL of Griess Reagent I and 50 μL of Griess Reagent II were added to both standard and sample wells. The absorbance at 540 nm was measured using an xMark microplate reader, and NO content in CCEC samples was calculated based on the standard curve.

### Kits to detect ROS (S0033S, Beyotime, China) levels

Cells were inoculated into 6-well plates ahead of time and wall-adhered to 80%, the wall-adherent cells were digested, and cell suspension was prepared. The ROS fluorescent probe was added to re-suspend the precipitated cells, mixed well, and incubated at 37 °C for 45 minutes. The supernatant was centrifuged and removed, washed three times using PBS to remove the probe that did not enter the cells, and finally, the fluorescence was measured at 488 nm excitation wavelength and 525 nm emission wavelength, and the relative fluorescence intensity was calculated based on the control group.

### Kits to detect MDA (S0131S, Beyotime, China) levels

Cells were inoculated into 6-well plates ahead of time, wall-adhered to 80%, wall-adherent cells were digested, centrifuged at 10 000 rpm for 10 minutes at 4 °C, and the sample supernatant was taken. Protein quantification was done using the BCA kitI (PC0020, Solarbio Science, China). According to the instructions, the storage solution, working solution, and 1, 2, 5, 10, 20, and 50 μM standard solutions were prepared sequentially. These solutions were then added into different EP tubes as required, mixed thoroughly, heated in a boiling water bath for 15 minutes, then cooled to room temperature, and finally centrifuged at 10 000 g for 10 minutes. Then, 200 μL of the supernatant was sucked up by a pipette gun and then added to 96-well plates, and the absorbance was measured at 532 nm. Finally, the MDA content was calculated from the standard curve and absorbance.

### Animals

All animal experimental protocols are approved by the Laboratory Animal Ethics Committee of Xinjiang Medical University (IACUC approval No. 20180222-60). Sprague–Dawley rats (male, 5 weeks, 200 ± 10 g) were provided by the Animal Experiment Center of Xinjiang Medical University and exposed to a regular 12/12-h light/dark cycle in plenty of water and food. After 2 weeks of adaptive feeding, 6 rats were randomly selected to form the normal control group (labeled NC), and the remaining 26 rats were identified as the diabetic group. Rats in the NC group were injected with an equal amount of sodium citrate buffer (0.1 mol/L, pH 4.5), and rats in the diabetic group were injected intraperitoneally with streptozotocin (45 mg/kg, S0130, Sigma, USA) for 2 consecutive days. After 2 days, blood was collected from the tail vein and random blood glucose was measured. When the blood glucose of the rats was >16.7 mmol/L, the rats were identified as diabetic models. After 4 times of apomorphine experiments and paired experiments at weeks 2, 4, 6, and 8, the rat model of DMED was established. The DMED model rats were randomly divided into 4 groups (6 rats each): the DMED group, the Yimusake group (treated with Yimusake by gastric gavage, 250 mg/kg, Xinjiang Hetian Uyghur Pharmaceutical Co, Ltd; labeled Y), the NLRP3 inhibitor group (received the NLRP3-specific inhibitor MCC950 via intraperitoneal injection, 10 mg/kg, MedChemExpress, USA; labeled MCC950), and the combined NLRP3 inhibitor and Yimusake group (treated with a combined of MCC950 (10 mg/kg) and Yimusake (250 mg/kg), labeled MCC950 + Y). These are the same animals from the previous publication (DOI: 10.1016/j.heliyon.2024.e38626).

### Immunohistochemical staining

The rat penile tissue was routinely sectioned, deparaffinized, and subjected to antigen retrieval. Primary antibodies including rabbit anti-NF-κB p65 (1:100, BA0610, Boster, China) were added and incubated overnight at 4 °C. Subsequently, goat anti-rabbit IgG antibody (catalog number PV9001, Beijing Zhongshan Golden Bridge Biotechnology Co., Ltd.) was introduced and incubated at room temperature for 30 minutes on the following day. DAB chromogenic solution (ZLI9019, Beijing Zhongshan Golden Bridge Biotechnology Co., Ltd.) was added for color development. Subsequently, the tissue sections were dehydrated, clearing using a series of alcohol and xylene gradients, followed by examination under a conventional optical microscope to evaluate the expression of NF-κB p65 in the rat penile tissue and capture images. Image J image analysis software was used for result analysis.

### Quantitative real-time PCR analysis

Total RNA was extracted from penile tissue and CCECs using the Trizol reagent (catalog number 15596026, Thermo Fisher Scientific, USA) and subjected to reverse transcription to generate cDNA using the SimpliAmp PCR thermal cycler (SimpliAmp, Thermo Fisher Scientific, USA). Subsequently, *NLRP3, NF-κB p65,* and *VEGFa* mRNA expression levels were assessed via RT-qPCR in the QuantStudio 1 Plus system (Thermo Fisher Scientific, USA). The 2^-ΔΔCT^ method was employed based on the obtained CT values. Detailed primer sequences can be found in Table 1.

**Table 1:** Sequence of primers.

Gene	Forward (5′-3′)	Reverse (5′-3′)
*NLRP3*	GATAGGTTTGCTGGGATA	GGTGTAGCGTCTGTTGAG
*NF-κB*	AGGCTTCTGGGCCTTATGTG	TGCTTCTCTCGCCAGGAATAC
*VEGFa*	CGACAGAAGGGGAGCAGAAA	GCTGGCTTTGGTGAGGTTTG
*β-Actin*	TGACAGACTACCTCATGAAGATCC	GCAACATAGCACAGCTTCTCTTTA

### Western blot analysis

Proteins were extracted from penile tissue and CCECs using RIPA lysis buffer (R0010, Solarbio, China) supplemented with PMSF (P0100, Solarbio, China). Protein quantification was performed according to the instructions of the BCA protein concentration determination kit. After that, 10 μL of protein per sample was loaded, and the proteins underwent SDS-PAGE using the electrophoresis system (PowerPac, Bio-RAD, USA) for 150 minutes, then were transferred to PVDF membranes (ISEQ00010, Millipore, USA) using a Trans-Blot for 120 minutes, followed by blocking with 5% milk (BS102, Lanjie Ke Technology, China) for 120 minutes. Subsequently, rabbit anti-eNOS (1:1000, 27 120-1AP, Proteintech, China), CD31 (1:1000, AF6191, Affinity, China), ET-1 (1:1000, DF6125, Affinity, China), VEGFa (1:1000, 19 003-1-AP, Proteintech, China), SOD-1 (1:1000, AF5198, Affinity, China), NOX1(1:1000, DF8684, Affinity, China), NOX2 (1:1000, DF6520, Affinity, China), NLRP3 (1:1000, BA3677, Boster Biological, China), NF-κB p65 (1:1000, BA0610, Boster Biological, China), p-NF-κB p65 (1:1000, AF2006, Affinity, China), IκBα (1:1000, AF5002, Affinity, China), p-IκBα (1:1000, AF2002, Affinity, China), IL-18 (1:1000, DF6525, Affinity, China), and β-actin (1:6000, AF7018, Affinity, China) were added and incubated overnight at 4 °C. After washing with TBST (T1085, Solarbio, China), the membranes were subjected to a 120-minute incubation with goat anti-rabbit IgG antibody (dilution 1:10 000, catalog number ZB2301, Beijing Zhongshan Golden Bridge Biotechnology Co., Ltd.). Upon completion of color development and image acquisition, Image J software (National Institutes of Health) was employed to quantify the grayscale values of the target and reference proteins, facilitating the determination of their respective ratios.

### Statistical analysis

Data were subjected to statistical analysis using SPSS version 26.0 (IBM, Armonk, NY, USA), with multiple group comparisons conducted via one-way analysis of variance (ANOVA). Quantitative data were statistically described employing the mean ± standard deviation format, and statistical significance was determined at *P* < .05 threshold. Graphical representations were crafted utilizing GraphPad Prism version 8.0 (GraphPad Prism 8.0, GraphPad Software, San Diego, CA, USA).

## Results

### Observation and identification of CCECs

**Figure 1:**
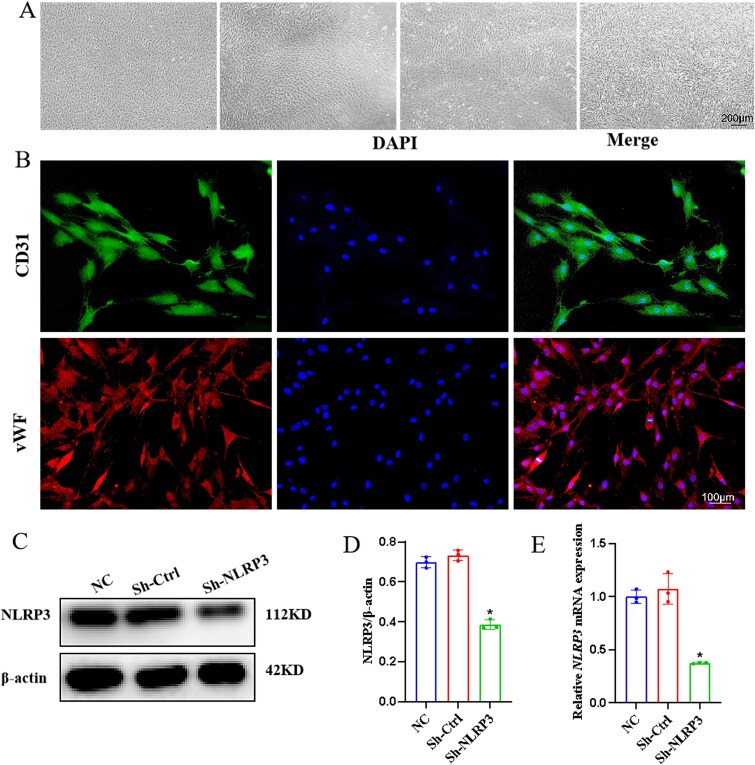
The observation and authentication of CCECs as well as the assessment of Sh-NLRP3 transfection efficacy. (A) Light microscopy examination of primary cultured CCECs at 100× magnification. (B) Immunofluorescence detection of CD31 and vWF protein expression in CCECs at 200× magnification. (C) Western blotting was used to determine the expression of the NLRP3 proteins of each group. (D) Relative expression of NLRP3 protein, ^*^P < .05 compared with the normal control group. (E) Expression status of NLRP3 mRNA, ^*^P < .05 compared with the normal control group.

Primary cultured CCECs were examined under a light microscope, revealing that endothelial cells in the first, second, and third generations displayed characteristic “cobblestone-like” clustered growth. By the fourth generation, a subset of cells exhibited elongation and displayed diverse morphologies (Figure 1A). Immunofluorescence technology was utilized to identify the expression of CD31 (green) and vWF (red) in the primary cultured CCECs (Figure 1B), with nearly all cells showing positive expression, indicating that the cell purity was suitable for subsequent experiments. Following lentiviral transfection of CCECs, we confirmed the success of transfection using RT-qPCR and Western blot. The results demonstrated a decrease in NLRP3 protein (Figure 1C and D) and NLRP3 mRNA expression in the Sh-NLRP3 group (*P* < .05) (Figure 1E), indicating successful transfection for future experiments.

### The impact of Yimusake on CCECs

**Figure 2:**
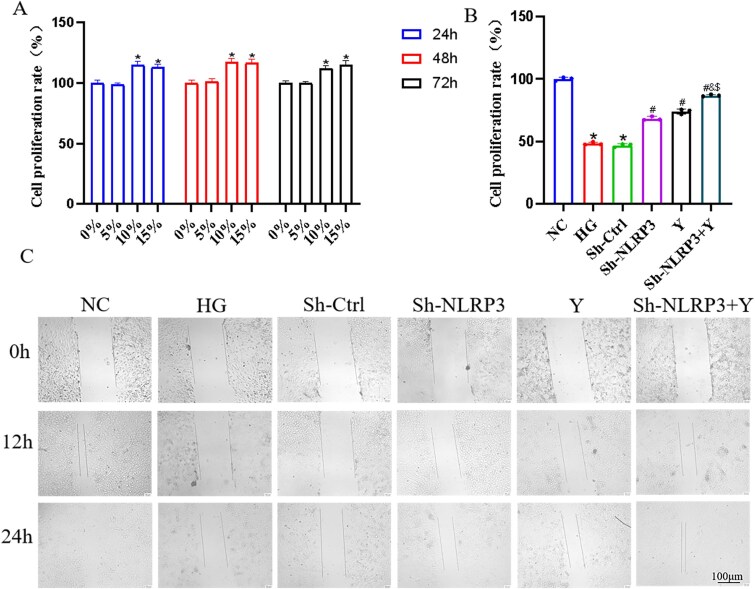
Yimusake intervention can enhance the vitality and migratory ability of CCECs. (A) Optimal screening of Yimusake drug–containing serum, ^*^P < .05 compared with the 0% group. (B) Proliferation rate of CCECs, ^*^P < .05 compared with the NC group; ^#^*P* < .05 compared with the HG group; ^&^*P* < .05 compared with the Sh-NLRP3 group; ^$^*P* < .05 compared with the Y group. (C) Migration experiment of CCECs.

Yimusake drug–containing serum was extracted from normal rats treated with Yimusake, and the MTT assay was utilized to determine the optimal serum concentration. The results indicated that compared to the NC group, the group cultured with 5% serum did not exhibit significant differences at 24, 48, and 72 hours (*P* > 0.05). In contrast, groups with 10% and 15% serum concentrations demonstrated varying degrees of stimulation on cell proliferation (*P* < .05). This suggests that 5% serum has the least impact on the growth of CCECs, leading to its selection as the optimal concentration for subsequent experiments, with a preferred intervention duration of 24 hours (Figure 2A). Subsequently, we utilized the MTT assay to assess the vitality of cells in each group. The results revealed that compared to the NC group, the HG group CCECs exhibited decreased vitality (*P* < .05). However, following the knockdown of NLRP3 and intervention with Yimusake, the vitality of CCECs in the Sh-NLRP3, Y, and Sh-NLRP3 + Y groups showed some recovery, with the Sh-NLRP3 + Y group displaying even more significant results (*P* < .05) (Figure 2B). Additionally, a cell scratch assay was conducted to evaluate migration capabilities. Initially, at 0 hours, no significant differences were observed among the groups. However, at 12 and 24 hours, the HG group displayed reduced migration rates, whereas the Sh-NLRP3, Y, and Sh-NLRP3 + Y groups experienced increased migration rates, with the shNLRP3 + Y group showing the most significant improvement (*P* < .05, Figure 2C). These findings underscore the ability of Yimusake intervention to enhance both the vitality and migration capabilities of CCECs.

### The impact of Yimusake on the NLRP3 inflammasome–mediated NF-κB signaling pathway *in vitro*

**Figure 3:**
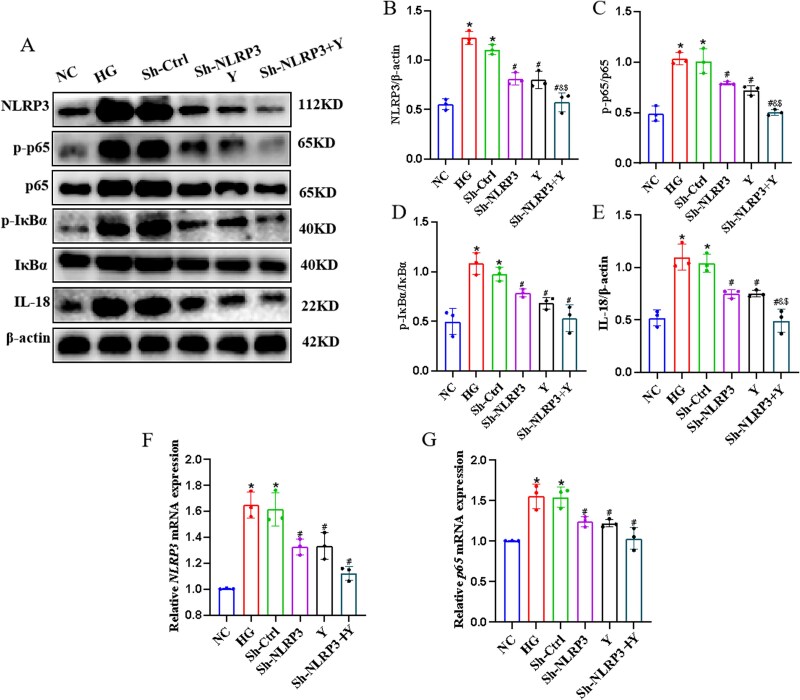
Yimusake can inhibit the expression of the NLRP3 inflammasome-mediated NF-κB signaling pathway *in vitro*. (A) Western blotting was used to determine the expression of the NLRP3, p65, p-p65, IκBα, p-IκBα, and IL-18 protein expression in CCECs of each group. (B) Relative expression of NLRP3. (C) Relative expression of p-p65/p65. (D) Relative expression of p-IκBα/IκBα. (E) Relative expression of IL-18 (F) PCR detection of *NLRP3* mRNA expression in CCECs. (G) RT-qPCR detection of *p65* mRNA expression in CCECs, ^*^P < .05 compared with the NC group, ^#^*P* < .05 compared with the HG group, ^&^*P* < .05 compared with the Sh-NLRP3 group, ^$^*P* < .05 compared with the Y group (*P* < .05).

To further validate our hypothesis, we extracted CCECs and administered serum containing Yimusake, as well as lentivirus-mediated knockdown of NLRP3, to study the changes in the expression of downstream factors. The results revealed that in the HG group, the expression of NLRP3 and its downstream factors, including p65, p-p65, IκBα, p-IκBα, and IL-18 increased (*P* < .05). Conversely, in the Sh-NLRP3, Y and Sh-NLRP3+ Y groups, the expression of NLRP3 and its downstream factors exhibited a decreasing trend (*P* < .05), with the Sh-NLRP3 + Y group showing more significant changes (*P* < .05), Among them, the expression of *NLRP3* and *p65* mRNA was reduced in the Sh-NLRP3 + Y group compared with the Sh-NLRP3 and the Y group, but the difference was not statistically significant (Figure 3A-G).

### The impact of Yimusake on oxidative stress *in vitro*

**Figure 4:**
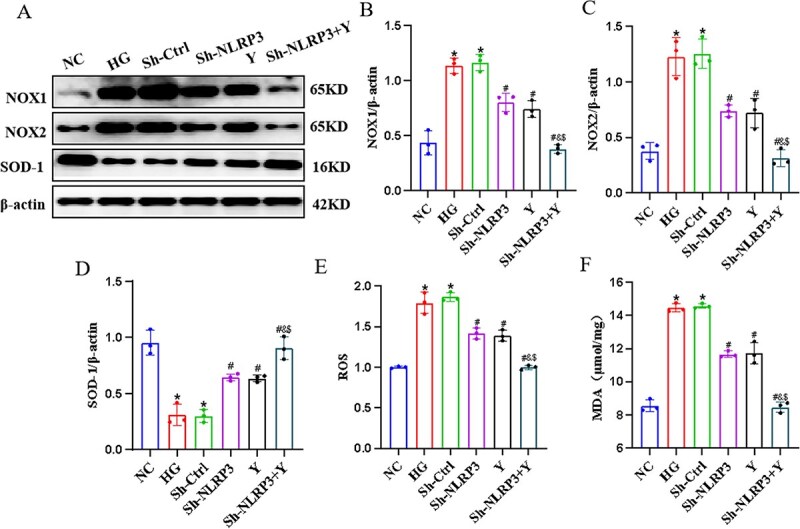
Yimusake can inhibit oxidative stress *in vitro*. (A) Western blotting was used to determine the expression of the NOX1, NOX2, and SOD-1 protein expression in CCECs of each group. (B) Relative expression of NOX1. (C) Relative expression of NOX2. (D) Relative expression of SOD-1. (E) Kit detection of ROS expression in CCECs. (F) Kit detection of MDA expression in CCECs, ^*^P < .05 compared with the NC group, ^#^*P* < .05 compared with the HG group, ^&^*P* < .05 compared with the Sh-NLRP3 group, ^$^*P* < .05 compared with the Y group (*P* < .05).

To verify the effect of Yimusake on oxidative stress, we investigated the changes of oxidative stress-related factors in extracted CCECs. The results showed that the expression of NOX1, NOX2, ROS, MDA was elevated and the expression of SOD-1 was decreased in the HG group compared to the NC group (*P* < .05), and the expression of NOX1, NOX2, ROS, MDA was decreased and the expression of SOD-1 was elevated in the Sh-NLRP3 group, Y group and Sh-NLRP3 + Y group compared to the HG group (*P* < .05) and was more significant in the Sh-NLRP3 + Y group (*P* < .05) (Figure 4A-F).

### The impact of Yimusake on endothelial function *in vitro*

**Figure 5:**
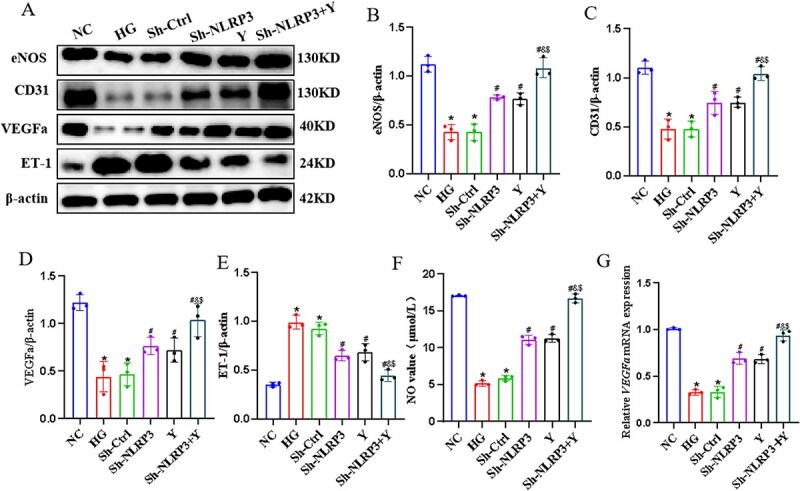
Yimusake can improve endothelial function *in vitro*. (A) Western blotting was used to determine the expression of the eNOS, CD31, VEGFa, and ET-1 protein expression in CCECs of each group. (B) Relative expression of eNOS. (C) Relative expression of CD31. (D) Relative expression of VEGFa. (E) Relative expression of ET-1. (F) Kit detection of NO expression in CCECs. (G) RT-qPCR detection of *VEGFa* mRNA expression in CCECs, ^*^P < .05 compared with the NC group, ^#^*P* < .05 compared with the HG group, ^&^*P* < .05 compared with the Sh-NLRP3 group, ^$^*P* < .05 compared with the Y group (*P* < .05).

Finally, we verified the effect of Yimusake on endothelial function in extracted CCECs, and the results showed that ET-1 expression was elevated and eNOS, CD31, VEGFa, and NO expression were decreased in the HG group compared to the NC group (*P* < .05), whereas after knockdown of NLRP3 and Yimusake interventions, the Sh-NLRP3 group, the Y group, and the Sh-NLRP3 + Y groups, ET-1 expression was decreased and eNOS, CD31, VEGFa, and NO expression was elevated (*P* < .05), and it was more significant in the Sh-NLRP3 + Y group (*P* < .05) (Figure 5A-G).

Assessment of erectile function and metabolic evaluation in rats can be found in the article by Zhu Bingbing,^12^ a member of our research team.

### The impact of Yimusake on the NLRP3 inflammasome–mediated NF-κB signaling pathway *in vivo*

**Figure 6:**
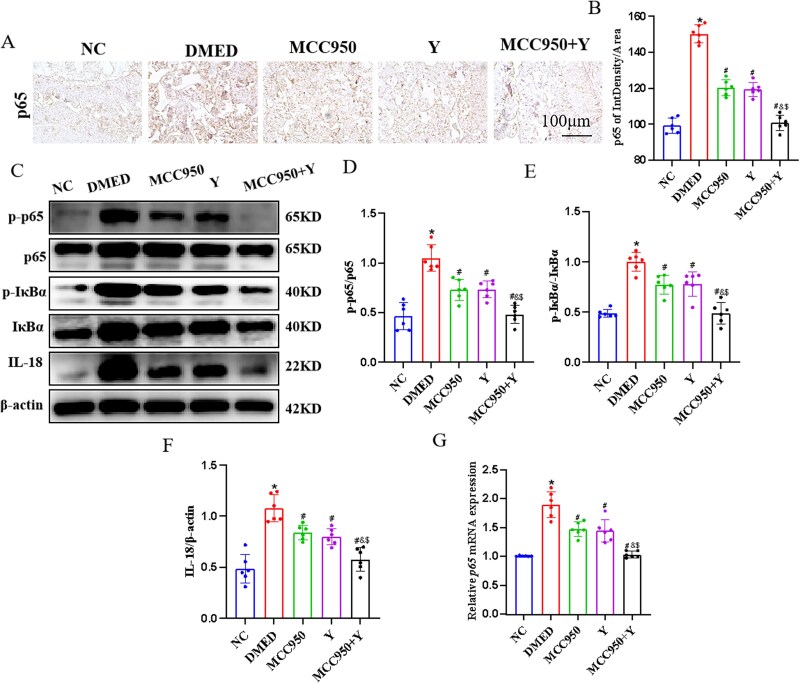
Yimusake can inhibit the expression of the NLRP3 inflammasome-mediated NF-κB signaling pathway in vivo. (A) Immunohistochemical detection of NF-κB p65 protein expression in rat penile tissue (x200). (B) Relative expression of p65. (C) Western blotting was used to determine the expression of the p65, p-p65, IκBα, p-IκBα, and IL-18 protein expression in rat penile tissue of each group. (D) Relative expression of p-p65/p65. (E) Relative expression of p-IκBα/IκBα, (F) relative expression of IL-18. (G) RT-qPCR detection of *p65* mRNA expression in rat penile tissue, ^*^P < .05 compared with the NC group, ^#^*P* < .05 compared with the DMED group, ^&^*P* < .05 compared with the MCC950 group, ^$^*P* < .05 compared with the Y group.

To validate our hypothesis that Yimusake could suppress NLRP3 inflammasome activation and its downstream factors, we studied the changes of NLRP3 downstream factors after downregulating NLRP3 and intervention with Yimusake. Our findings revealed that in the DMED group, the expression levels of NLRP3 downstream factors including p65, p-p65, IκBα, p-IκBα, and IL-18 were elevated significantly (*P* < .05). Conversely, in the MCC950, Y, and MCC950 + Y groups, a downward trend was observed in the expression of NLRP3 downstream factors (*P* < .05), with the combined intervention group demonstrating more pronounced alterations (*P* < .05) (Figure 6A-G). The above results indicate that Yimusake can inhibit the expression of the NLRP3 inflammasome-mediated NF-κB signaling pathway, thus confirming our hypothesis.

The impact of Yimusake on oxidative stress *in vivo* can be found in the article by Zhang Rui,^20^ a member of our research team.

### The impact of Yimusake on endothelial function *in vivo*

**Figure 7:**
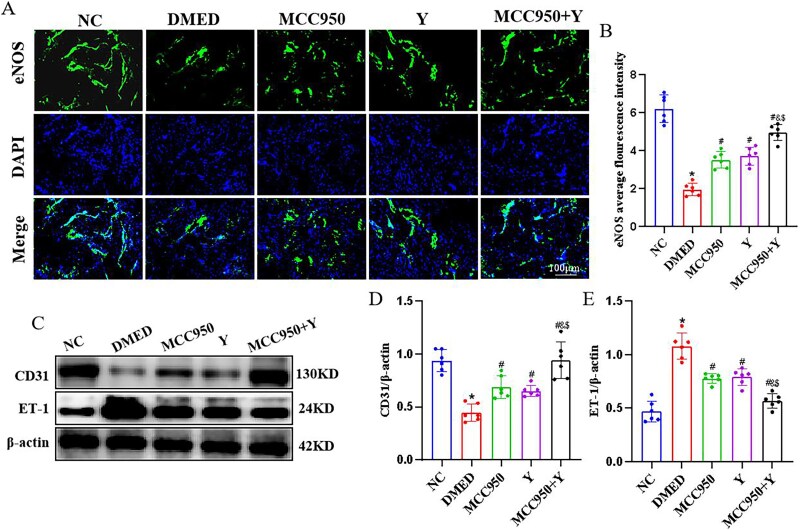
Yimusake intervention can ameliorate endothelial function. (A) Immunofluorescence detection of eNOS protein expression in penile tissue (×200). (B) Western blotting was used to determine the expression of the CD31 and ET-1 protein expression in the penile tissue of each group. (C) Relative expression of CD31. (D) Relative expression of ET-1. (E) Relative expression of eNOS, ^*^P < .05 compared with the NC group; ^#^*P* < .05 compared with the DMED group; ^&^*P* < .05 compared with the MCC950 group; ^$^*P* < .05 compared with the Y group.

Endothelial nitric oxide synthase plays a crucial role in facilitating penile erection, while Endothelin-1 (ET-1) can promote vasoconstriction of the penile corpus cavernosum, leading to DMED. The results demonstrate that in the DMED group, the expression of eNOS and CD31 proteins decreased (*P* < .05), while the expression of ET-1 protein increased (*P* < .05). Conversely, in the MCC950, Y, and MCC950+ Y groups, the expression of eNOS and CD31 proteins increased (*P* < .05), while the expression of ET-1 protein decreased (*P* < .05), with the MCC950+ Y group showing more significant changes (*P* < .05) (Figure 7A-E). The above results indicate that Yimusake intervention can improve endothelial cell function.

## Discussion

Diabetes mellitus–induced erectile dysfunction is a prevalent yet frequently overlooked complication among diabetic patients. Research indicates that as diabetes progresses, the risk of both microvascular and macrovascular complications incrementally escalates, heightening the risk of DMED^21^ and potentially triggering more severe cardiovascular and cerebrovascular diseases.^22^ Consequently, investigating effective preventative and therapeutic strategies is of paramount importance.

**Figure 8:**
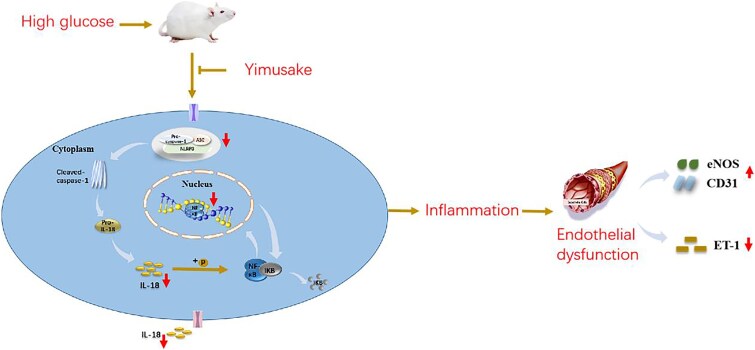
Schematic of the study. Yimusake intervention can downregulate the NLRP3 inflammasome–mediated NF-κB signaling pathway, thereby preventing the expression of inflammatory cytokines and subsequently ameliorating endothelial dysfunction.

Yimusake, a traditional herbal remedy from Xinjiang, has been reported in previous studies to exhibit therapeutic benefits through significantly reducing the latency of erection, enhancing libido, and improving erectile function (Figure 8).^23^ This was also demonstrated in our group’s preliminary study.^13^ To investigate the mechanism of action, our initial experiments detected body weight, water intake, food intake, blood glucose, and glycated hemoglobin levels in rats but found no significant differences between intervention groups.^12^ These results indicate that Yimusake’s improvement of erectile function in DMED rats is unrelated to diabetes amelioration or blood glucose reduction. Based on this finding,^12^ this study further conducted experiments on the basis of previous research and found that Yimusake and MCC950 (a specific NLRP3 inflammasome inhibitor) exerted similar therapeutic effects on ED, suggesting that Yimusake may delay ED progression by suppressing NLRP3 inflammasome activation. These results also suggest that there is a potential association between Yimusake’s mechanism and NLRP3 inflammasome regulation. Therefore, this study aims to further explore whether Yimusake improves penile vascular endothelial function in the treatment of DMED by inhibiting the NF-κB signaling pathway mediated by the NLRP3 inflammasome.

Research has indicated that the NLRP3 inflammasome plays a pivotal role in the development of ED^24^ and that inhibiting the NLRP3 inflammasome-mediated NF-κB signaling pathway can significantly attenuate the inflammatory response in diabetic nephropathy.^25^ Sun et al. pointed out that the levels of inflammatory factors, including NLRP3 and NF-κB, are elevated in human umbilical vein endothelial cells following high glucose injury.^26^ To further elucidate the mechanism of action of Yimusake in the DMED rat model and the high glucose–treated CCECs model, we observed a decrease in the expression of key molecules involved in the NLRP3 inflammasome–mediated NF-κB signaling pathway (NLRP3, IL-18, IκBα, p-IκBα, NF-κB p65, p-NF-κB p65) following Yimusake tablet intervention, which was consistent with the results after MCC950 intervention. These findings suggest that Yimusake may suppress the inflammatory response by inhibiting the NLRP3 inflammasome–mediated NF-κB signaling pathway. Furthermore, the combined intervention of Yimusake and MCC950 resulted in more significant effects, suggesting that the inhibition of the NLRP3 inflammasome–mediated NF-κB signaling pathway by Yimusake is only one of multiple mechanisms through which it improves ED.

A penile erection involves a series of vascular smooth muscle relaxation responses regulated by neuroendocrine mechanisms. The cavernous body of the penis, serving as the effector organ for penile erection, plays a pivotal role in the development and progression of ED, contingent upon the integrity of its vascular endothelial function.^27^ Vascular endothelial growth factor (VEGF) is the most specific and pro-angiogenic growth factor acting on endothelial cells, playing a key role in both physiological and pathological angiogenesis.^28^ Among the VEGF family members, VEGFa is the most extensively studied.^29^ Studies demonstrated that VEGFa expression was significantly lower in ED patients and rats compared to normal controls.^30^ Additionally, VEGFa enhanced the proliferation of corpus cavernosum smooth muscle cells and endothelial cells, thereby improving erectile function.^31^ Endothelial nitric oxide synthase is predominantly expressed in endothelial cells, with the ability to reduce endothelial cell adhesion, regulate vascular tone, and promote penile erection.^32^ Conversely, ET-1 is recognized as one of the most potent vasoconstrictors identified to date. It is primarily produced by vascular endothelial cells and exerts a strong contractile effect, playing a crucial role in maintaining the flaccid state of the penis.^33^ CD31, an essential marker of endothelial cells, plays a critical role in maintaining endothelial cell continuity.^34^ Studies by Yin et al. revealed a significant decrease in the expression of CD31 and eNOS, along with an elevation in the expression of the inflammatory factor IL-6, and a notable occurrence of apoptosis in endothelial cells in STZ-induced DMED rats.^35^ Additionally, other studies have shown that overexpression of NLRP3 can stimulate the abundant production of ET-1, impairing erectile function.^36^ Our experimental findings demonstrated a decrease in the expression of VEGFa, eNOS, and CD31 and an increase in ET-1 expression in the penile tissues of DMED rats. However, following individual interventions with MCC950 and Yimusake, both groups exhibited a significant increase in VEGFa, eNOS, and CD31 expression, coupled with a notable decrease in ET-1 expression. This trend was also observed in the *in vitro* high-glucose-induced CCECs model, indicating that inhibiting the inflammatory response can facilitate the restoration of endothelial cell function. Notably, the combined intervention group showed even more pronounced effects, suggesting that Yimusake may alleviate DMED by directly enhancing endothelial function, consistent with the findings of Hou et al.^37^

From the above experiments, we found that endothelial cell damage is directly related to DMED. In a hyperglycemic state, a large amount of accumulated ROS will cause inflammatory damage to vascular endothelial cells by regulating the expression of inflammation-related factors, thus leading to vascular endothelial dysfunction.^38^ It is evident that oxidative stress may play an important role in inducing endothelial cell injury. Reactive oxygen species are a class of molecules with important biological functions in living organisms, and abnormally elevated levels are associated with a variety of pathological processes.^39^ As a product of lipid oxidation, malondialdehyde (MDA) is one of the markers of oxidative stress injury, accumulating with oxidative stress, and its elevated levels directly correlate with cellular damage severity.^40^ It was found that an increase in ROS production leads to an increase in NLRP3-mediated ET-1 expression, which induces erectile dysfunction.^36^ Li^41^ found that the level of oxidative stress could be attenuated and apoptosis of cavernous tissues could be reduced by inhibiting the expression of MDA, which led to the improvement of erectile function in diabetic rats. In this study, we found that the expression of ROS and MDA was elevated in the CCECs injury model established by *in vitro* incubation with high glucose and was reduced after the intervention of Yimusake. The same trend was observed in the penile tissues of rats in the DMED group.^19^ NOXs are the main source of ROS generation, and their role in oxidative stress, inflammation, fibrosis, and tumour development makes them popular targets in relevant therapeutic areas, especially the two family members, NOX1 and NOX2.^42^ The activity level of SOD, the main antioxidant enzyme in the cell, is a key factor in the evaluation of the important indicator of the function of endogenous oxygen radical scavenging system.^43^ Studies have shown^44^ that in the DMED rat model, protein expression of NOX1 and NOX2 was elevated, while SOD expression levels were decreased. Decreasing the expression of NOX1 and NOX2 improved ED in diabetic mice.^45^ In this study, we showed that NOX1 and NOX2 expression was elevated and SOD-1 expression was decreased in the DMED group in the CCECs injury model established by *in vitro* incubation with high glucose, and NOX1 and NOX2 expression was decreased and SOD-1 expression was elevated after the intervention of Yimusak, and the effect of the combination of the intervention groups was more significant. The same trend was also observed in the penile tissue of DMED rats.^19^ The above studies suggest that erectile dysfunction in DMED rats is related to endothelial dysfunction caused by elevated levels of oxidative stress; however, Yimusak can inhibit the level of oxidative stress and improve endothelial function, thus restoring erectile function.

Although the present study confirmed that Yimusake attenuates inflammatory responses and oxidative stress, improves penile vascular endothelial function, and promotes erectile tissue repair by inhibiting the NLRP3 inflammasome–mediated NF-κB signaling pathway, several limitations remain to be addressed. For example, the significantly enhanced efficacy observed in the combination therapy group compared to the single-drug group suggests that Yimusake may act through additional pharmacological mechanisms, such as modulation of the TNF-α-mediated inflammatory pathway.

## Conclusion

DM can enhance oxidative stress and aggravate endothelial damage in the corpus cavernosum by activating the NLRP3 inflammasome–mediated NF-κB signaling pathway. In contrast, Yimusake can inhibit oxidative stress and mitigate endothelial damage by downregulating this pathway, thereby facilitating the restoration of the erectile tissue in rats with DMED.

## Supplementary Material

ARRIVE-Supporting_Information_qfaf079
