# Supplementary material for: Yimusake ameliorates corporal endothelial dysfunction by down-regulating the NLRP3 inflammasome–mediated NF-κB signaling pathway and inhibiting oxidative stress
Source: Sex Med. 2025 Oct 13;13(5):qfaf079. doi: 10.1093/sexmed/qfaf079 (PMC12516956; doi:10.1093/sexmed/qfaf079)
Supplement: ARRIVE-Supporting_Information_qfaf079 [file arrive-supporting_information_qfaf079.doc]

**Supplementary File**

Item 2b：

In cell experiments, each group should have at least three replicate wells, and the experiment should be repeated three times independently. In animal experiments, each group should have six rats. This sample size meets the statistical power requirements for one-way analysis of variance and complies with the minimum sample size requirements of the laboratory animal ethics committee (to avoid overuse of animals).

Item 4b：

Animal cages were randomly assigned to avoid concentrating animals from the same experimental group in the same area (e.g., cages were randomly distributed across different levels of the animal housing facility);

The order of experimental procedures (e.g., drug administration, testing) was determined using a random number table to avoid systematic errors caused by differences in procedure timing;

In cell experiments, cells from the same batch were randomly assigned to different experimental groups to ensure consistency in culture conditions (e.g., position within the incubator, passage time).

Item 5：

A single-blind method was used during the experimental implementation phase:

Animal grouping information was coded by independent experimenters, and the personnel administering the drugs only knew the code numbers and did not know the specific groups (such as the DMED group, Yimusake intervention group, etc.).

Item 6b：

The primary outcome measure of this study was the expression level of eNOS protein in cavernous endothelial cells (CCECs), which was used to determine the sample size. eNOS is a key marker of endothelial function, and changes in its expression directly reflect the degree of improvement in endothelial function. In preliminary experiments, it was confirmed to be sensitive to interventions (Yimusake or NLRP3 inhibitor), meeting the selection criteria for the primary outcome measure.

Item 7b：

Verify the normality of the data using the Shapiro-Wilk test;

Verify the homogeneity of variance among groups using the Levene test;

If the data does not follow a normal distribution or the variances are not homogeneous, replace the one-way analysis of variance (ANOVA) with a nonparametric test (such as the Kruskal-Wallis H test) and clearly indicate this in the results.

Item 9c：

Animals were housed in the SPF-grade environment of the Animal Experiment Centre at Xinjiang Medical University, with a temperature of 22±2°C, humidity of 50±5%, and a 12-hour light-dark cycle (lighting time 8:00-20:00). After a 2-week acclimatisation period, the experiment began to ensure that the animals were adapted to the environment.

Item 9d：

High glucose (30 mM) stimulation was used to simulate the high glucose environment of diabetes, inducing damage to CCECs (concentration settings for the high glucose damage model in Reference 5);

STZ (45 mg/kg) intraperitoneal injection is a classic method for establishing a type 1 diabetes model (its efficacy was validated in Reference 12);

Yimusake dosage (250 mg/kg) and MCC950 dosage (10 mg/kg) were determined based on prior studies and pre-experimental results to ensure intervention efficacy without significant toxicity.

Item 10a：

The descriptive statistics of each group are as follows (taking key indicators as examples):

In cell experiments, the relative expression levels of eNOS protein were 1.12±0.08 in the NC group, 0.43±0.78 in the HG group, 0.43±0.86 in the Sh-Ctrl group, 0.78±0.24 in the Sh-NLRP3 group, 0.76±0.60 in the Y group, and 1.08±0.10 in the Sh-NLRP3+Y group, respectively.

In animal experiments, the relative expression levels of eNOS protein were 6.22±0.72 in the NC group, 1.96±0.33 in the DMED group, 3.51±0.43 in the MCC950 group, 3.72±0.47 in the Y group, and 4.95±0.42 in the MCC950+Y group, respectively.

Item 10b：

Effect Size and Confidence Interval (Taking the Upregulating Effect of Yimusake on eNOS as an Example)

In cell experiments, compared with the HG group, the effect size of eNOS expression in the Y group was 0.76 (95% Confidence Interval: 0.18-0.49).

In animal experiments, compared with the DMED group, the effect size of eNOS expression in the Y group was 4.40 (95% Confidence Interval: 1.24-2.29).
